# The effect of adenoid hypertrophy on maxillofacial development: an objective photographic analysis

**DOI:** 10.1186/s40463-016-0161-3

**Published:** 2016-09-20

**Authors:** Cigdem Fırat Koca, Tamer Erdem, Tuba Bayındır

**Affiliations:** 1 Otorhinolaryngology (KBB) Department, Malatya State Hospital, Malatya, Turkey; 2 Medical Faculty, Otorhinolaryngology (KBB) Department, Acıbadem University, Istanbul, Turkey; 3 Medical Faculty, Otorhinolaryngology (KBB) Department, Inonu University, Malatya, Turkey

**Keywords:** Adenoid hypertrophy, Mouth breathing, Photographic analysis, Facial morphology

## Abstract

**Background:**

Deformity in the dental arc and facial skeleton by adenoid hypertrophy due to chronic mouth breathing is a well-known process. Most of the related studies have been based on cephalometric analyses. The aim of this study is to detect the presence of skeletal deformities on the soft tissue by analyzing distances and angles on photographs.

**Methods:**

Ninety-seven children having between 25 and 100 % of adenoids, ages 4–12 years (48 boys, 49 girls), and 90 cases having 0–25 % adenoid tissue, ages 4–12 years (54 boys, 36 girls), were studied by clinical history, physical examination (including endoscopy), and standardized clinical photographs. The children and parents were asked if any of the following were present in the children: snoring, sleep apnea, daytime sleepiness, poor school performance, mouth breathing during sleep, smoking parents, and restlessness during sleep.

**Results:**

The assessment of linear and angular measurements on the clinical photographs showed, in the group having thicker adenoids compared with controls, a statistically significant increase in the distance between nasion and tip and nasion and subnasale and in the angle between Frankfort horizontal plane-gnathion-angulus mandible; there was also a statistically significant decrease in the distance between endocanthion and exocanthion and the angles between tragion-angulus mandible and gnathion and between nasion-angulus mandible and gnathion.

**Conclusions:**

The analyses showed a significant increase in the anterior face height and increase in the angle between Frankfort horizontal plane-gnathion-angulus mandible and a retropositioned and posterior-rotated mandible due to thicker adenoids.

**Trial registration:**

2010/140 Date: 04 January 2010.

## Background

Adenoid hypertrophy is the most common pathology causing upper airway obstruction in childhood [1]. Adenoid hypertrophy causes upper airway obstruction and may affect both dental and maxillofacial development [2]. Nasal breathing is partially obstructed due to large adenoids, and this leads to mouth breathing and typical “adenoid face” [3]. Adenoid face is characterized as having upper lip incompetence, a retropositioned hyoid bone, a narrow upper dental arch, retropositioned mandibular incisors, increased anterior face height, a narrow or “V”-shaped maxillary arch, increased mandibular plane angle, and a posterior-rotated mandible in comparison with healthy controls [2]. The positioning of the tongue is downward due to mouth breathing, and the balance between the tongue and mandible is different in comparison with healthy children. This leads to the lower positioning of the mandible. In this situation, a number of postural changes can occur, such as open mandible posture and extension of the head. Intraorally, a narrow maxillary arch, high palatal arch, and class 2 or 3 dental malocclusion may be seen. Cephalometrically, a large anterior face height and increased mandibular plane angle can be noted [3]. Timing of the removal of excessive adenoid tissue is important for the recovery of the craniofacial growth pattern [2]. After adenoidectomy and facilitation of nasal breathing, accelerated mandibular growth and closure of the mandibular plane angle have been reported. Nasal airway obstruction may lead to some problems, such as night discomfort, behavioral issues, poor school performance, and daytime sleepiness [3].

## Methods

### Ethics consent and permissions

We studied 97 children having between 25 and 100 % of adenoids, ages 4–12 (48 boys, 49 girls), and 90 cases having 0–25 % adenoid tissue, ages 4–12 (54 boys, 36 girls), who consulted the Department of Otolaryngology Head and Neck Surgery, Inonu University, between November 2010 and March 2011. We started the study after approval of Human Ethics Committee of Inonu University (2010).

After defining the process in detail to the parents, the children (whose parents approved) were included in the study. A detailed, complete otolaryngological examination was performed. The information of all children was registered by a previously prepared anamnesis form.

### The exclusion criteria

The exclusion criteria were maxillofacial surgery, dysmorphism and craniofacial syndromes, septal deviation, chronic illnesses, decongestant use, upper airway obstruction causes other than adenoid hypertrophy, acute upper airway infection, thumb sucking, and tonsillar hypertrophy.

### Patients, clinical and instrumental evaluations

**Fig. 1:**
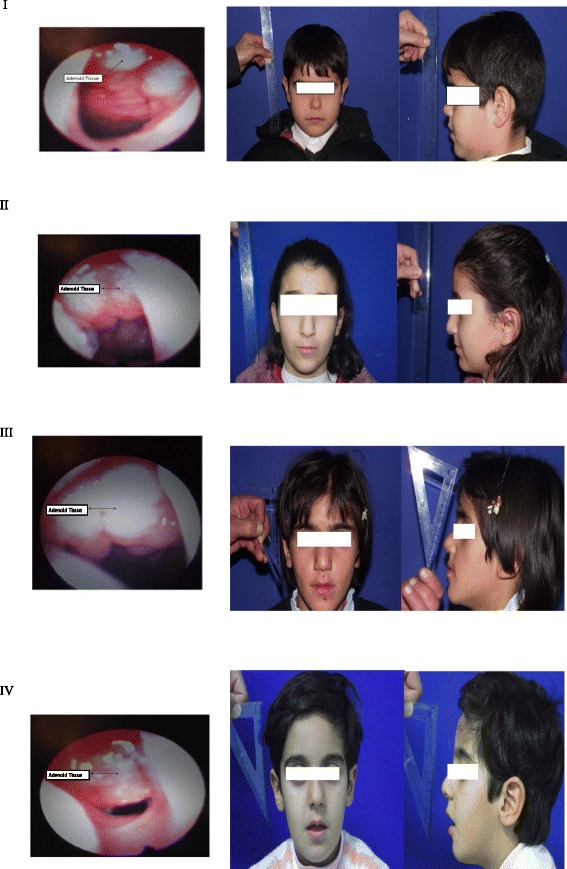
I: 0–25 % adenoid tissue (Group 1: controls). II: 25–50 % adenoid tissue (Group 2a). III: 50–75 % adenoid tissue (Group 2b). IV: 75–100 % adenoid tissue (Group 2c) and the actual representative pictures of each group

The children having 0–25 % adenoids were declared as Group 1 (controls: 90 children). The children having between 25 and 100 % of adenoids (97 children) were declared as Group 2 and were divided into three subgroups according to the degree of their adenoid tissue obstruction in choana. Group 2a: 25–50 % adenoid tissue (50 children), Group 2b: 50–75 % adenoid tissue (35 children), and Group 2c: 75–100 % adenoid tissue (12 children). Flexible fiberoptic nasopharyngolaryngoscopy (FNFL) was used to evaluate the degree of obstruction of the adenoid tissue for each child. We saved all flexible fiberoptic nasopharyngolaryngoscopic images of all children (Fig. 1).

Both the children and their parents were asked if any of the following were present in the children: snoring, sleep apnea, daytime sleepiness, poor school performance, mouth breathing during sleep, smoking parents, and restlessness during sleep. We compared whether the children had these complaints, and each complaint was scored by the visual analog scale (VAS). We included the children having these complaints lasting at least for 6 months. The average period was 1.06 years (6 months to 4 years).

Photographs of Groups 1 and 2 were taken using a point-and-shoot or SLR-like digital camera with a 90-mm lens (Fujifilm finepix s2500 HD 12.2 megapixels Fujinon 18× optical zoom lens) for standardized facial plastic measurements from a 150-cm distance. Children were instructed to position their heads in a natural head position as much as possible. The lateral photographs were taken with the Frankfort horizontal plane parallel to the ground. In both photographs, the ruler was held in equal distance between the facial plane and photograph machine. Anterior and lateral photographs of children in both groups were taken with a ruler, and maxillofacial measurements were performed on the standardized clinical photographs. Data of both groups were registered and compared. The Scion Image (Beta 4.02 Win version; Scion Corporation, Frederick, MD, USA.) software program was used for measurements of the distances between reference points by referring to the 1 cm shown on the ruler in anteroposterior photographs. The measurements of the angles between points given were performed on the lateral view photographs. This method was previously used in two facial analysis studies performed in our clinic [4, 5].

### The reference points

The reference points used for the measurements included the vertex (v; the top of the head), trichion (tr; midpoint of the anterior hairline), glabella (g; the most forward-projecting point of the forehead in the midline of the supraorbital ridges), nasion (n; midpoint of the nasofrontal suture), tip (t: the junction of the inferior margin of the nasal ridge and the columella), subnasale (sn; the middle point of the inferior border of the nasal columella), right and left ala nasi (ala; the most lateral point of nasal alar wall), gnathion (gn; in the midline, the lowest point on the lower border of the chin), endocanthion (en; the point at which the inner ends of the upper and the lower eyelid meet), exocanthion (ex; the point at which the outer ends of the upper and the lower eyelid meet), zygion (zy; zygomatic process), angulus oris (an: the lateral limit of the oral fissure), stomion (st: the median point of the oral slit when the lips are closed), superaurale (sa; top of the auricular helix), subaurale (sba; the lowest point of the ear), pogonion (pg: the most forward-projecting point on the anterior surface of the chin), infraorbitale (io: the point below the orbit; it is a guide point for the Frankfort horizontal plane), angulus of mandible (anm: the angle formed by the inferior border of the mandible and the posterior edge of the ramus of the lower jaw), and tragion (tra: the superior margin of the tragus of the ear). Distances between those reference points were measured in millimeters.

### Distances and angles

**Fig. 2:**
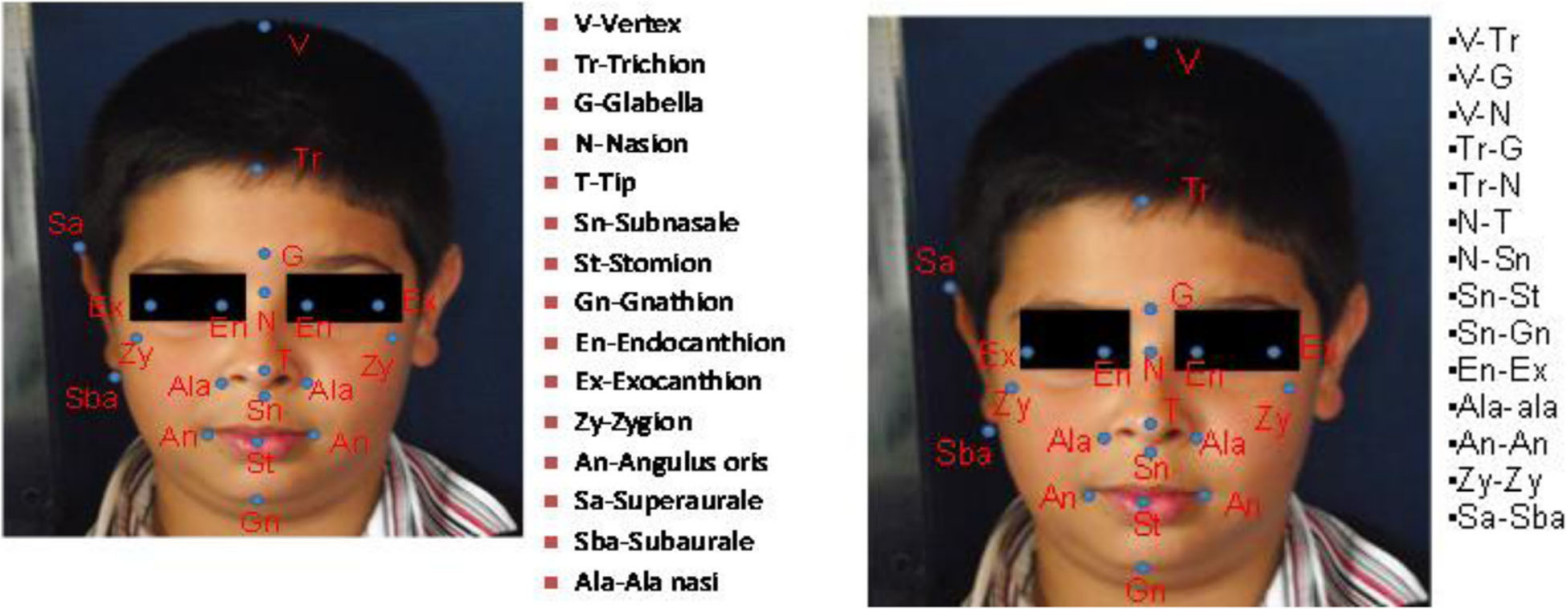
Facial-analyzing points. V:Vertex, Tr:Trichion, G:Glabella, N:Nasion, T:Tip, Sn:Subnasale, St:Stomion, Gn:Gnathion, En: Endocanthion, Ex: Exocanthion, Zy: Zygion, An: Angulus oris, Sa: Superaurale, Sba: Subaurale, Ala: Ala nasi and distances measured on anterior photographs

**Fig. 3:**
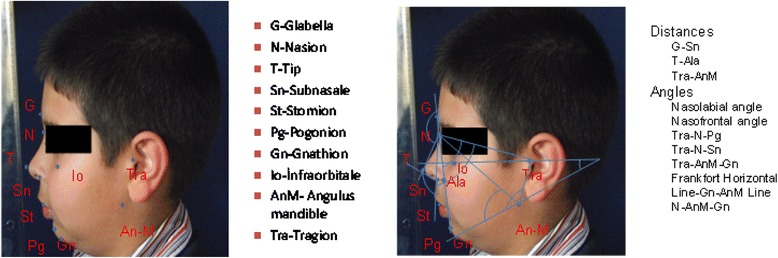
Lateral photographs and facial-analyzing points, angles, and distances

We measured the distances between vertex and trichion (v-tr), vertex and glabella (v-g), vertex and nasion (v-n), trichion and glabella (tr-g), trichion and nasion (tr-n), nasion and tip (n-t), nasion and subnasale (n-sn; the nose length), subnasale and stomion (sn-st), subnasale and gnathion (sn-gn; the height of the lower third of the face), endocanthion and exocanthion (en-ex; eye fissure width), right and left ala nasi (ala-ala; width of the nose), angulus oris and angulus oris (an-an; width of the mouth), zygion and zygion (zy-zy; width of the face), superaurale and subaurale (sa-sba; ear length), glabella and subnasale (g-sn; the length of the middle third of the face), tip and ala (t-ala), and tragion and angulus mandible (tra-anm) and the angles between tragion, nasion, and pogonion (tra-n-pg), tragion, nasion, and subnasale (tra-n-sn), tragion, angulus mandible, and gnathion (tra-anm-gn), Frankfort horizontal plane (a line extending from the most inferior point of the orbital margin to the left tragion), gnathion, and angulus mandible (frank-gn-anm), nasion, angulus mandible, and gnathion (n-anm-gn), and nasolabial and nasofrontal angles (Figs. 2 and 3).

### Statistical analyses

The statistical analyses were performed with SPSS version 16 for Windows. Measurable variables were defined as average ± standard deviation (SD), and discontinuous data were defined as numbers and percentages. The normal distribution of measurable variables was carried out by Shapiro–Wilk normality test. We used Pearson’s chi-squared test and Fisher’s exact test for discontinuous data and unpaired *T*-test, one way variance analysis (ANOVA) test, and least significant difference method (LSD) for measurable variables. Pearson correlation test was used to evaluate the relation between variables. The significance level *p* < 0.05 was chosen.

## Results

The average values of the distances (v-tr), (v-g), (v-n), (tr-g), (tr-n), (n-t), (n-sn), (sn-st), (sn-gn), (en-ex), (ala-ala), (an-an), (zy-zy), (sa-sba), (g-sn), (t-ala), and (tra-anm) and the angles (tra-n-pg), (tra-n-sn), (tra-anm-gn), (frank-gn-anm), and (n-anm-gn), SDs, *t*-test values, and *p* values are given in Tables 1 and 2.

**Table 1:** Distance measurements between groups (*p < 0.05) (*n*: number of children in each group)

Distances (mean ± SD) (mm)	Group 1 (*n* = 90)	Group 2a (*n* = 50)	Group 2b (*n* = 35)	Group 2c (*n* = 12)	*P* value
vertex and trichion (v-tr)	*49.9* ± 9.9	48,7 ± 11,1	52,5 ± 12,4	51,2 ± 10,3	0.435
vertex and glabella (v-g)	106 ± 14	105,8 ± 16	110,2 ± 15	103,8 ± 9,9	0.421
vertex and nasion (v-n)	123.1 ± 14.1	120,3 ± 15,2	124,8 ± 16,4	118,9 ± 9,2	0.415
trichion and glabella (tr-g)	56,9 ± 8,5	57,5 ± 8,3	58,3 ± 9,4	53,7 ± 6,1	0.431
trichion and nasion (tr-n)	73,4 ± 10,1	72,4 ± 8,6	71,3 ± 11,6	68,8 ± 6,7	0.421
nasion and tip (n-t)	36,7 ± 5,8	39,4 ± 6,6	39,7 ± 7,6	37,9 ± 5,4	0.035^*^
nasion and subnasale (n-sn)	*46,8* ± 5,9	49,5 ± 6,4	48,2 ± 7,1	46,9 ± 5,8	0.101
subnasale and stomion (sn-st)	23,8 ± 2,1	21,6 ± 3,1	21,5 ± 3,3	20,2 ± 2,8	0.741
subnasale and gnathion (sn-gn)	59,2 ± 8,2	60,3 ± 8,9	58,9 ± 8,3	55,4 ± 10,6	0.373
endocanthion and exocanthion (en-ex)	31 ± 3,1	30,9 ± 3,2	30,3 ± 3,3	28,3 ± 2,5	0.036^*^
ala and ala (ala-ala)	36,7 ± 4,2	36,4 ± 4,3	35,9 ± 3,9	33,8 ± 3,1	0.137
angulus oris and angulus oris (an-an)	48 ± 6,2	48,1 ± 7	46,7 ± 7,3	43,5 ± 5,6	0.116
zygion and zygion (zy-zy)	100,1 ± 13,2	101,5 ± 8,1	100,5 ± 9,4	96,7 ± 6,1	0.590
superaurale and subaurale (sa-sba)	56,4 ± 5,6	54,4 ± 9,2	56,5 ± 6,6	53 ± 5,4	0.174
glabella and subnasale (g-sn)	57,3 ± 6,3	59 ± 5,8	56,2 ± 6	58,2 ± 5	0.196
tip and ala (t-ala)	23 ± 2,7	23,5 ± 3,1	23,7 ± 2,4	21,6 ± 2,9	0.121
tragion and angulus mandible (tra-anm)	53,5 ± 7,1	52,5 ± 6,1	51 ± 7,2	50,7 ± 7,6	0.237

**Table 2:** Angle measurements between groups (*p < 0.05)

Angles (mean ± SD)	Group 1 (*n* = 90)	Group 2a (*n* = 50)	Group 2b (*n* = 35)	Group 2c (*n* = 12)	*P* value
tragion, nasion and pogonion (tra-n-pg)^0^,	65,8 ± 5,1	68,1 ± 5,2	67,1 ± 4,8	65,9 ± 3,1	0.074
and tragion, nasion and subnasale (tra-n-sn)^0^	75,7 ± 5,4	77,4 ± 4,7	77,1 ± 5,5	74,7 ± 3,4	0.150
tragion, angulus mandible and gnathion (tra-anm-gn)^0^	136,8 ± 7,7	135,1 ± 5,6	134 ± 8,9	131,1 ± 5,2	0.035^*^
Frankfort horizontal plane, gnathion and angulus mandible (frank-gn-anm)^0^	28 ± 4	28 ± 2,8	28,9 ± 3,5	31 ± 3,9	0.043^*^
nasion, angulus mandible and gnathion (n-anm-gn)^0^	67,1 ± 5,4	64,3 ± 5,7	64,1 ± 6,1	63,9 ± 9,2	0.011^*^

There was a statistically significant increase in the distance between nasion-tip (*p* = 0.035) and the angle between Frankfort horizontal plane-gnathion-angulus mandible (*p* = 0.043) and statistically significant decrease in the distance between endocanthion-exocanthion (*p* = 0.036) and the angles between tragion-angulus mandible-gnathion (*p* value: 0.035), nasion-angulus mandible-gnathion (*p* = 0.011), in the group having thicker adenoids compared with controls.

There was a statistically significant increase in the distance between nasion-subnasale (n-sn) in the children having higher adenoids (Group 2) than in controls (Group 1). The average length was 48.7 mm (±6.6 mm) in Group 2 and 46.8 mm (±5.9 mm) in the control group (*p* = 0.038, *p* < 0.05). In the evaluation between the four groups (Group 1: 0–25 % adenoid tissue (control group), Group 2a: 25–50 % adenoid tissue, Group 2b: 50–75 % adenoid tissue, and Group 2c: 75–100 % adenoid tissue), the distance between nasion-subnasale was longer in the groups having thicker adenoids compared with controls, but there was no statistical difference (*p* = 0.101)(*p* > 0.05). Statistically, there was no difference in age and gender between groups. The mean age was 7.9 ± 2.4 in Group 2 and 8.0 ± 2.5 in control Group 1. There was no significant difference in gender between groups, according to chi-Squared statistical test (*p* > 0.05).

There were statistically significant differences in vas1 (vas score of snoring; *p* = 0.0001), vas2 (vas score of mouth breathing in sleep; *p* = 0.0001), vas3 (vas score of sleep apnea; *p* = 0.006), vas6 (vas score of poor school performance; *p* = 0.001) between groups. In Group 2, the complaints of the degree of snoring, sleep apnea, and mouth breathing was greater than in the control group. In addition, there was poor school performance in Group 2 (Table 3). We compared the smoking ratio in parents for children between Groups 1 and 2 (Table 4).

**Table 3:** Statistical analyses of age and vas between groups 1 and 2

	Group (*n*)	mean	Std. Deviation	*t*-test value	*p*- value
Age	1.00 (90)	8.0000	2.59645	−0.249	0.804 ***p*** **> 0.05**
	2.00 (97)	7.9072	2.49617		
**vas1**	1.00 (90)	1.5000	2.63618	4.400	0.0001 ***p*** **< 0.05**
	2.00 (97)	3.5258	3.55344		
**vas2**	1.00 (90)	1.4444	2.63985	5.341	0.0001 ***p*** **< 0.05**
	2.00 (97)	3.8969	3.53696		
**vas3**	1.00 (90)	0.2333	0.98357	2.767	0.006 ***p*** **< 0.05**
	2.00 (97)	0.9072	2.10695		
vas4	1.00 (90)	0.1444	0.78699	1.084	0.280 *p* > 0.05
	2.00 (97)	0.3093	1.22781		
vas5	1.00 (90)	0.5889	1.85360	3.525	0.273 *p* > 0.05
	2.00 (97)	1.9588	3.22722		
**vas6**	1.00 (90)	0.4778	1.55229	3.719	**0.001** ***p*** **< 0.05**
	2.00 (97)	1.7526	2.88686		

vas1: vas score of snoring, vas2: vas score of mouth breathing while sleeping, vas3: vas score of sleep apnea, vas4: vas score of daytime sleepiness, vas5: vas score of restless sleep, and vas6: vas score of poor school performance

**Table 4:** Smoking ratio of parents between groups 1 and 2

GROUP		No smoking	1 Parent smoking	2 Parents smoking	Total	Chi-squared test value	*p* value
1	number	50	18	22	90	10.564	0.005^*^
	Within group %	55.6 %	20.0 %	24.4 %	100.0 %		
2	number	32	23	42	97		
	Within group %	33.0 %	23.7 %	43.3 %	100.0 %		

In statistical analyses, the smoking ratio of parents in Group 2 was higher than in control Group 1 (*p* = 0.005)

*: *p* value for the smoking ratio of parents in Group 2

## Discussion

Adenoid hypertrophy is the most common pathology causing upper airway obstruction in childhood [1]. Patients experience mouth breathing as a result of nasal obstruction. Therefore, incisors, which have large dental pulp, are exposed to moisture and get cold by the effect of evaporation. The cold causes pain, and this triggers the tongue-pushing reflex in an effort to warm the incisors. If the mouth breathing continues, tongue pushing and anterior positioning of the tongue also continue, which this leads to an anterior open-bite deformity [6]. An increase in the anterior face height may be seen due to nasal obstruction caused by chronic mouth breathing [7, 8].

Woodside and Linder-Aronson reported the increase in anterior face height in nasal-obstructed individuals by aging. They also pointed out that the anterior face height is independent from other skeletal units, and it depends on only factors that affect mandibular posture, such as mouth breathing [9]. Fields and colleagues identified a relation between dentofacial morphology and nasal airway capacity [10]. Yamada and colleagues found inferior and posterior mandible, superior and posterior development of the condil, and anterior crossbite deformity due to nasopharyngeal obstruction [11]. In our study, we measured 17 distances and 7 angles on the anterior and lateral standardized clinical photographs of normal children and those with thicker adenoids. There was a statistically significant increase in the distance between nasion-tip (*p* = 0.035) and the angle between Frankfort horizontal plane-gnathion-angulus mandible (*p* = 0.043) and a statistically significant decrease in the distance between endocanthion-exocanthion (*p* = 0.036) and the angles between tragion-angulus mandible-gnathion (*p* = 0.035) and nasion-angulus mandible-gnathion (*p* = 0.011) in the group having thicker adenoids compared with controls. There was a statistically significant increase in the distance between nasion-subnasale (n-sn) in Group 2 compared with Group 1. In children with adenoid hypertrophy, the positioning of the tongue is downward due to mouth breathing and the balance between the tongue and mandible is different in comparison with healthy children. This leads to the lower positioning of the mandible. In this situation, a number of postural changes can occur, including open mandible posture and extension of the head. Cephalometrically, a large anterior face height and increased mandibular plane angle can be observed [3].

In our study, the increase of the distance between the nasion-tip and the nasion subnasale (anterior face height) and an increase in the angle between the Frankfort horizontal plane and the angulus-gnathion mandible (craniomandibular angle) was similar to the findings presented in previous cephalometric studies [2, 3, 6–8, 12–14].

In our study, children were evaluated according to their school performance. School performance was scored according to the VAS. A statistically poorer school performance was observed in the group having thicker adenoids compared with controls (*p* < 0.05). This result was parallel to the literature. The failure in school performance may result from the relation between chronic nocturnal hypoxia and neurocognitive functioning. How the interruption of sleep causes neurobehavioral difficulties is unknown. It is probable that sleep interruption and episodic hypoxia cause propulsive functional failures due to changes in the neurochemical structure of the prefrontal cortex. Poor performance in linguistic skills and visual–spatial functions and poor academic performance confirm that children with adenoid hypertrophy have problems with their prefrontal cortical functions. A significant increase in school performance has been observed after adenoidectomy [15, 16].

Smoking status was asked of the parents of both groups. There were three results: no smoking, only one parent smoking, and both parents smoking. An increased smoking ratio was observed in Group 2 compared with Group 1. The results were statistically significant (*p* < 0.05). The relationship between cigarette smoke exposure and adenoid hypertrophy has been reported by many studies. The increase of nitric oxide concentration may cause cytotoxic effects. Cigarette smoke exposure increases the synthesis of heat shock proteins in tissues. Heat shock proteins are synthesized in tissues in response to environmental stressors. Cigarette smoke causes its destructive effects in adenoid tissue by reagent oxygen and nitrogen products. Heat shock protein 70 is responsible for adenoid hypertrophy in children exposed to cigarette smoke. Heat shock protein 70 prevents adenoid regression by preventing apoptosis in hypertrophied adenoid tissue [17].

Our analyses showed a significant increase in the anterior face height and an increase in the angle between the Frankfort horizontal plane and the angulus-gnathion mandible, as well as a retropositioned and posterior-rotated mandible, due to increased thickness in the adenoids. Treatment of nasal obstruction in growing children results in normal dentofacial development. For this reason, it is important to address the direct relation between nasal airway obstruction and dentofacial development, and early treatment should be administered to help to ensure normal development. We must familiarize ourselves with dentofacial development. Earlier detection is necessary to prevent adenoid face. Normalization of the breathing pattern has a favorable effect on dentofacial development. Pediatricians, orthodontists, and otolaryngologist should cooperate to ensure proper evaluation of nasal obstruction and dental abnormalities.

## Conclusions

In conclusion, the effects of adenoid hypertrophy on maxillofacial development have been analyzed by cephalometric studies up to now. However, the effects of adenoid hypertrophy on maxillofacial development have not been analyzed by a photographic method; in this respect, this is the first reported study. The analyses on clinical photographs were parallel to the cephalometric analyses reported in the literature, and our study also observed the reflection of skeletal deformities on soft tissue distances and angles.

## Abbreviations

ala: Right and left ala nasi
an: Angulus oris
anm: Angulus of mandible
en: Endocanthion
ex: Exocanthion
FNFL: Flexible fiberoptic nasopharyngolaryngoscopy
frank: Frankfort horizontal plane
g: Glabella
gn: Gnathion
io: Infraorbitale
n: Nasion
pg: Pogonion
sa: Superaurale
SA: Standard deviation
sba: Subaurale
sn: Subnasale
st: Stomion
t: Tip
tr: Trichion
tra: Tragion
v: Vertex
VAS: Visual analog scale
VAS: Visual analog scale
zy: Zygion

## References

[1] Çelik O, Yalçın Ş, İnan E, Kaygusuz İ, Yanık H. Adenoid hipertrofisinin maksillofasial gelişim üzerine etkileri. K.B.B. ve Baş Boyun Cerrahisi Dergisi. 1995;3:222-6.

[2] Arsalah R, Waheed H, Fatima J (2009). Cephalometric assessment of patients with adenoidal faces. J Pak Med Assoc.

[3] Peltomaki T (2007). The effect of mode of breathing on craniofacial growth. Eur J Orthod.

[4] Erdem T, Ozturan O (2008). Objective measurement of the deviated nose and a review of surgical techniques for correction. Rhinology.

[5] Erdem T (2010). Long-term effectiveness of projection control suture in rhinoplasty. Rhinology.

[6] Mizrahi E (1978). A rewiev of anterior open bite. Br J Orthod.

[7] Aronson SL (1979). Respiratory function in relation to facial morphology and the dentition. Br J Orthod.

[8] Mahony D, Karsten A, Aronson SL (2004). Effects of adenoidectomy and changed mode of breathing on incisor and molar dentoalveolar heights and anterior face heights. Aust Orthod J.

[9] Janson GRP, Metexas A, Woodside DG (1994). Variation in maksillary and mandibular molar and incisor vertical dimension in 12-year-old subjects with excess, normal and short lower anterior face height. Am J Orthod Dentofacial Orthop.

[10] Fields HW, Warren DW, Black K, Phillips CL (1991). Relationship between vertical dentofacial morphology and respiration in adolescents. Am J Orthod Dentofacial Orthop.

[11] Yamada T, Tanne K, Miyamoto K, Yamauchi K (1997). Influences of nasal respiratory obstruction on craniofacial growth in young Macaca fuscata monkeys. Am J Orthod Dentofacial Orthop.

[12] Zucconi M, Caprioglio A, Calori G, Strambi LF, Oldani A, Castronovo C (1999). Craniofacial modifications in children with habitual snoring and obstructive sleep apnoea: a case-control study. Eur Respir J.

[13] Zettergren LW, Forsberg CM, Aranson SL (2006). Changes in dentofacial morphology after adeno-/tonsillectomy in young children with obstructive sleep apnoea--a 5-year follow-up study. Eur J Orthod.

[14] Yamaguchi H, Sueshi K (2003). Malocclusion associated with abnormal posture. Bull Tokyo Dent Coll.

[15] Gottlieb DJ, Chase C, Vezina RM, Heeren TC, Corwin MJ, Auerbcah SH (2004). Sleep- disordered breathing symptoms are associated with poorer cognitive function in 5-year-old children. J Pediatr.

[16] Rhodes SK, Shimoda KC, Wald LR, O’Neil PM, Oexmann MJ, Collop NA (1995). Neurocognitive deficits in morbidly obese children with obstructive sleep apnea. J Pediatr.

[17] Ayçiçek A, Dilek H, Sargın R, Şahin O, Kenar F, Dereköy SÖ (2009). Heat shock protein 70 and inducible nitric oxide synthase expression in adenoid tissue of children exposed to passive smoke. Türkiye Klinikleri J Med Sci.

